# The performance of single and combination test strategies using visual inspection, cytology, high-risk HPV DNA and HPV16/18 to screen South African women with and without HIV-infection

**DOI:** 10.1186/s13027-024-00586-3

**Published:** 2024-05-09

**Authors:** Greta Dreyer, Cathy Visser, Gerrit Jan Dreyer, Matthys H. Botha, Frederick H. van der Merwe, Karin L. Richter, Leon C. Snyman

**Affiliations:** 1 Department of Obstetrics and Gynaecology, Faculty of Health Sciences, University of Pretoria, Pretoria, South Africa; 2 Department of Statistics and Actuarial Science, Faculty of Economic and Management Sciences, Stellenbosch University, Stellenbosch, South Africa; 3 Department of Obstetrics and Gynaecology, Faculty of Health Sciences, Stellenbosch University, Stellenbosch, South Africa; 4 Department Medical Virology, Faculty of Health Sciences, University of Pretoria, Pretoria, South Africa

**Keywords:** Triage test, Cervical cancer, Cervical cancer screening strategy, HIV-infection, Screening test performance

## Abstract

**Background:**

Cervical cancer screening strategies should ideally be informed by population-specific data. Strategies recommended for secondary prevention, are often inadequately studied in populations with high cervical disease burdens. This report describes the test performance measured against CIN2 + /CIN3 + histology in HIV-positive women (HPW) and HIV-negative women (HNW) with the aim to determine the most effective strategies to identify South African women at risk.

**Methods:**

Primary screening using visual inspection, cytology and HPV DNA (cobas®) was performed in two South African provinces on 456 HPW and 639 HNW participating in the multicentric DiaVACCS trial. Histology was obtained for 91.7% screen-positive and 42.7% screen-negative participants, and unavailable histology was determined by multiple imputation to adjust for verification bias. Cross-sectional test performance was calculated for single and combination test strategies with and without intermediate risk categories using different cut-offs. Minimum acceptability for sensitivity and specificity, treatment and follow-up numbers were considered to evaluate strategies.

**Results:**

The only single test to reach acceptability in HPW was cytology (LSIL) [sensitivity 71.2%; specificity 90.5%; treatment 33.4%]; in HNW only HPV (hr) qualified [sensitivity 68.2%; specificity 85.2%; treatment 23.5%]. The universally best performing strategy which also resulted in smaller treatment numbers without intermediate risk group was primary HPV(hr), with treatment of both HPV(16/18) and cytology (ASCUS +) [HPW: sensitivity 73.6%; specificity 89.7%; treatment 34.7%. HNW: sensitivity 59.1%; specificity 93.6%; treatment 13.9%].

DNA testing for hrHPV (any) and hrHPV (16/18) was the best universally acceptable strategy with an intermediate risk category (early follow-up) in HPW [sensitivity 82.1%; specificity 96.4%; treatment 17.1%; follow-up 31.4%] and HNW [sensitivity 68.2%; specificity 96.7%; treatment 7.6%; follow-up 15.9%]. In comparison, using both HPV (16/18) and cytology (ASCUS +) as secondary tests in hrHPV positive women, decreased follow-up [HPW 13.8%, HNW 9.6%], but increased treatment [HPW 34.7%, HNW 13.9%].

**Conclusion:**

Using hrHPV (any) as primary and both HPV16/18 and cytology as secondary tests, was universally acceptable without an intermediate risk group. Strategies with follow-up groups improved screening performance with smaller treatment numbers, but with effective management of the intermediate risk group as prerequisite.

**Supplementary Information:**

The online version contains supplementary material available at 10.1186/s13027-024-00586-3.

## Background

The high estimated age standardised incidence rate of cervical cancer among South African women (35.3/100 000) relays the epidemiology of high-risk human papilloma virus (HPV) [1]. Local data show a high prevalence of HIV-infection, HPV-infection and pre-invasive disease, with increase over time [2–5]. This local epidemiology relates to a largely unscreened population, untreated cervical disease, and an HIV-epidemic which was only controlled with antiretrovirals (ARVs) after many years [4].

Cervical cancer control in South Africa is therefore a critical health priority. The national cervical cancer control policy supports cytology and HPV-based primary screening with a long interval [6], but provincial implementation is insufficient, mostly offering opportunistic screening based on cytology [7]. Molecular screening has several advantages, and HPV-testing is known to reduce cervical cancer incidence and mortality [8, 9]. HPV screening is widely reported as highly sensitive, although control groups with histology results are often limited and most data originates from groups with low disease prevalence [10–12].

The recent World Health Organization (WHO) screening guidelines recommend HPV primary screening [13], but the comparative diagnostic performance of different screening options has not been sufficiently investigated in Sub-Saharan Africa [13]. Recent studies have focused on the screening test performance among HIV-positive women [14–18] with less known about performance among HIV-negative women in countries with a high HIV and HPV prevalence [19, 20]. Numerous studies tried to address the challenge of ideal triage for high-risk HPV (hrHPV) testing [21–25], but the optimal screening algorithm of test, triage, surveillance, and treatment must balance the local risk threshold with resource availability and must therefore be determined based on local data [13].

This study forms part of a larger screening trial, named DiaVACCS, performed by the Vaccine and Cervical Cancer Screen (VACCS) consortium to support the choice of screening, triage and treatment algorithms for South Africa. The study protocol was approved by the Faculty of Health Sciences Research Ethics Committees (FHS REC) of the University of Pretoria (196/2014) and Stellenbosch University (reciprocal approval 2015), registered as a clinical trial (ClinicalTrials.gov identification number NCT02956031) and conducted according to the principles of the Declaration of Helsinki. The University of Pretoria FHS REC is accredited nationally by the National Health Research Ethics Council of the South African Department of Health (REC-120208-018) and internationally by the Office of Human Research Protection of the USA Department of Health & Human Services (Federalwide Assurance FWA 00002567 and IRB 0000 2235 IORG0001762), while the respective registration numbers for Stellenbosch University are REC-130408-012, FWA 00001372 and IRB0005240.

The study design, population, methodology and baseline results have all been published and include high hrHPV and histology proven cervical intraepithelial neoplasia (CIN) rates in HIV-negative and HIV-positive screening populations [26]. The current analysis of the diagnostic performance of single and combined test strategies aims to provide evidence for the drafting of local guidelines with a practical and simple triage and treatment algorithm, with or without an intermediate-risk category.

## Materials and methods

Screen-eligible women aged 25 to 65 years, unscreened for 5 years or longer, were recruited at three study sites in Tshwane District, Kalafong Provincial Tertiary and Tygerberg Academic Hospitals. Data for two HIV-cohorts were separately collected and analysed, namely women living with HIV, this cohort called HIV-positive women (HPW) (n = 456), and women self-reported or tested to be HIV-negative (HNW) (n = 648). In accordance with South African national and WHO screening guidelines, women with unknown/undisclosed HIV-status (n = 9) were included as HNW [13, 27].

After obtaining informed consent, demographic and clinical data, cervical cytology was collected followed first by visual inspection using a solution of 3–5% acetic acid (VIA), then sampling for molecular analysis (cervical brush, Rovers; transported in Thinprep® PreservCyt, Hologic, and SurePath^TM^, BD), and lastly visual inspection was done using Lugol’s iodine solution (VILI). Visual inspection was reported as negative, positive/high grade or uncertain/low grade and analysed using uncertain/low grade as threshold. Cytology was reported using the Bethesda system and analysed according to thresholds of atypical squamous cells of undetermined significance (ASCUS) and low-grade squamous intraepithelial lesion (LSIL). For DNA testing we used the HPV cobas® test (Roche Molecular Systems) according to manufacturer’s instructions and used thresholds of “any hrHPV” and “HPV16/18” for analysis.

**Fig. 1:**
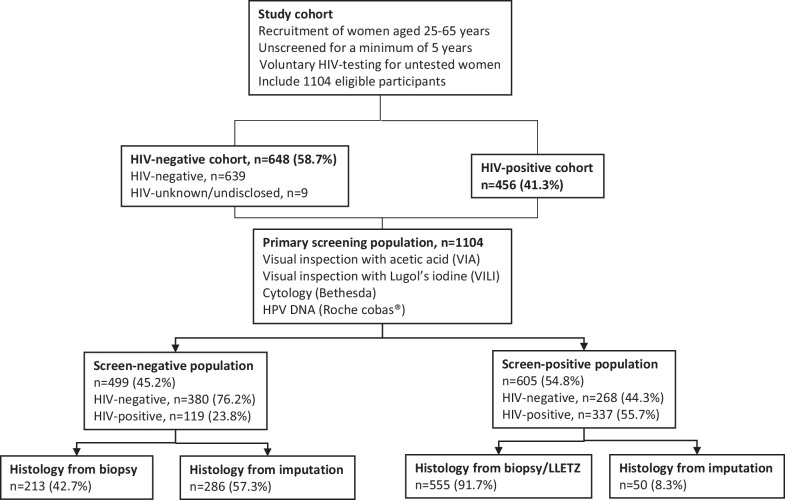
Flow diagram describing study population according to primary screening and histology results

Cervical biopsies were performed on 242 women with positive screening and on 213 screen-negative women. Large loop excision of the transformation zone (LLETZ) procedures were indicated for positive screening or diagnostic tests and were performed on 313 women. We used the worst grade of histology per participant as the final histologic diagnosis. The assumption that screen-negative women would have negative biopsies was found to be invalid and would create significant disease ascertainment bias. Verification biased adjusted (VBA) values for missing histology were therefore simulated based on age, HIV-status, use of ARVs, and screening results using multiple imputation (Language R; R foundation for Statistical Computing, Austria). We calculated test performance and standard error using these values [28]. Figure 1 describes the study population. The calculation method for the VBA values was previously published [26].

Microsoft Excel® 2016 (Microsoft Corp., Redmond, Washington, USA) was used for data recording, cleaning, and all other analyses. Prevalence values and the performance of single and combination test strategies against histology endpoints of CIN2 + and CIN3 + (sensitivity, specificity, positive and negative predictive values (PPV, NPV)) were calculated as proportions. False positivity rate (1-specificity) was defined as percentage screen-positive women with final histology < CIN2 and < CIN3; sensitivity for CIN2 + and CIN3 + was used to calculate negative predictive values. We interpreted test strategies mainly according to sensitivity and NPV for CIN3 + and specificity and PPV for < CIN2 histology; *p*-value < 0.05 was considered significant. Additional digital data files contain the 95% confidence intervals (95% CI).

Screening strategies included five single test strategies consisting of visual inspection, cytology, and HPV-testing analysed according to different thresholds for positivity as shown in brackets; all single tests resulted in two outcomes, namely high risk (positive test) and low risk (negative test). For the interpretation of test performance, thresholds for acceptability were selected due to limitations of using receiver operator characteristic (ROC) curve and area under the curve (AUC) for binary predictors [29]. In view of South Africa’s long screening interval, the best universally achievable sensitivity (68% for detecting CIN3 +) was selected as the standard of acceptability. In order to limit over-treatment (treatment of < CIN2) and over-burdening of the service, a minimum acceptable specificity of 85% for detecting CIN2 + was selected (corresponding to > 80% for detecting CIN3 +). Test combinations were modelled in pursue of this selected minimum specificity which would lead to no more than 15% overtreatment.

**Fig. 2:**
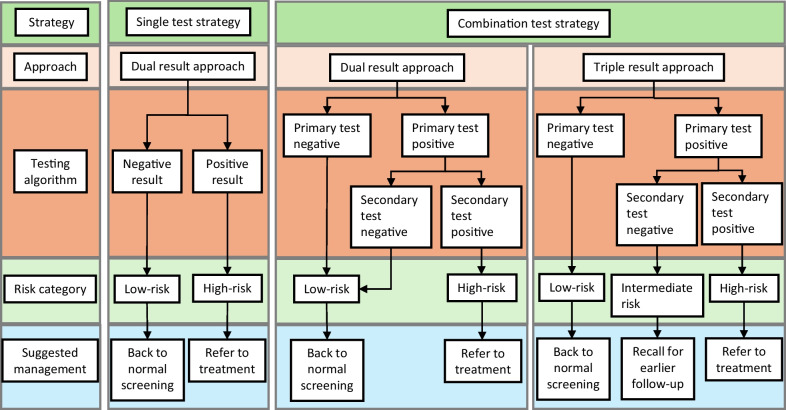
Modelling diagram describing test strategies, approaches and algorithms used to calculate screening performance

Seven combinations of the single tests, as well as the built-in HPV16/18 test were selected, for which the performances were then calculated using two approaches. Firstly, we calculated the outcomes of these seven test combinations resulting in a dual result of high risk (double positive) and low risk (all other results). Secondly, the test performance of the same combinations was calculated assuming a triple result of high risk (double positive), intermediate (primary test positive, secondary test negative) and low risk (primary test negative, secondary test not performed). See Fig. 2. In the triple result approach, the performance of combinations which could be done with either assessment as primary test, were calculated both ways, leading to 12 strategies. The assumption was made that only a positive (or invalid, cytology: n = 20) primary screening test was followed by a secondary test which could usually be reflex tested by the laboratory. Due to diverse usages of the term “triage”, we chose “secondary test” to limit confusion.

## Results

### Demographics and epidemiology

The mean age was 41.3 years, similar for the two HIV-groups; detailed HIV and epidemiological data were reported before [26]. Histology results were available for 768 women, confirming CIN3 + in 92 HPW (20.2% of total cohort; 26.1% of biopsied women; prevalence adjusted to 23.3% using VBA) and 51 HNW (7.9% of total cohort; 12.3% of biopsied; prevalence statistically adjusted to 10.2%). Cervical cancer was proven in 1.4% of women; VBA data suggest a 2% prevalence.

### Test performance

#### Single test strategies

The performance of single tests (A–E) is shown for detecting both CIN2 + and CIN3 + histology to enable comparison with published data (Table 1). Visual inspection performed poorly as screening tests and neither VILI nor VIA reached the selected acceptability standards (sensitivity for CIN3 + of 68%; specificity for CIN2 + of 85%) in any sub-population.

**Table 1:** Performance of single test strategies to predict CIN2 + and CIN3 + histology among HIV-positive and HIV-negative cohorts

HPW: HIV-positive women (n = 456)		Histology diagnosis used to calculate test performance							
Test (threshold)	Positivity rate %	CIN2 + histology CIN2 + Prevalence: 203/456 (44.5%)				CIN3 + histology CIN3 + Prevalence: 106/456 (23.3%); ICC prevalence: 13/456 (2.9%)			
Performance indicators	Refer to treatment	Sensitivity %	Specificity %	PPV %	NPV %	Sensitivity %	Specificity %	PPV %	NPV %
A: Visual inspection (VIA?)	47.8	66.5	67.2	61.9	71.4	75.5	60.6	36.7	89.1
B: Visual inspection (VILI?)	50.4	67.0	62.9	59.1	70.4	76.4	57.4	35.2	88.9
C: Cytology (ASCUS)	39.9	63.6	79.1	70.9	73.0	75.5	70.9	44.0	90.5
D: Cytology (LSIL)	33.4	55.7	90.5	83.0	71.1	71.2	82.0	54.8	90.3
E: hrHPV (any)	48.5	78.8	75.9	72.4	81.7	82.1	61.7	39.4	91.9

HNW: HIV-negative women (n = 648)		Histology diagnosis used to calculate test performance							
Test (threshold)	Positivity rate%	CIN2 + prevalence: 164/648 (25.3%)				CIN3 + prevalence: 67/648 (10.3%); ICC prevalence: 9/648 (1.4%)			
Performance indicators	Refer to treatment	Sensitivity %	Specificity %	PPV %	NPV %	Sensitivity %	Specificity %	PPV %	NPV %
A: Visual inspection (VIA?)	18.7	35.6	87.0	47.9	80.0	50.0	84.9	27.3	93.7
B: Visual inspection (VILI?)	20.9	35.6	84.1	43.0	79.5	51.5	82.6	25.2	93.8
C: Cytology (ASCUS)	17.0	41.7	91.3	61.8	82.3	59.1	87.8	35.5	95.0
D: Cytology (LSIL)	8.0	20.1	97.5	72.7	78.7	35.9	96.4	52.3	93.1
E: hrHPV (any)	23.5	49.1	85.2	52.6	83.3	68.2	81.6	29.6	95.8

CIN2 +  = cervical squamous intraepithelial neoplasia grade 2 or worse;

CIN3 +  = cervical squamous intraepithelial neoplasia grade 3 or worse;

ICC = invasive cervical cancer;

VIA? = visual inspection with acetic acid of uncertain or worse;

VILI? = visual inspection with Lugol’s iodine of uncertain or worse;

ASCUS = atypical squamous cells of unknown significance or worse;

LSIL = low grade squamous intra-epithelial lesion or worse;

any = any one or more of the 14 specified high-risk HPV DNA types

95% Confidence intervals are shown in the supplementary files

In both cohorts the most sensitive test was hrHPV (any) (HPW: 82.1%; HNW: 68.2%), and the most specific was cytology (LSIL) (HPW: 90.5%; HNW: 97.5%). Neither cytology, nor HPV-testing had acceptable accuracy to be used as single screening test universally, i.e. for both HIV-groups. In the HIV-positive cohort only cytology (LSIL) reached the acceptability standards, with referral rate of 33.4% (95% CI 29.0–37.8). In the HIV-negative cohort only hrHPV (any) reached both standards; referral rate 23.5% (95% CI 20.2–26.7).

#### Combination test strategies involving primary and secondary tests

The calculation of these strategies, the risk groups and suggested management is discussed in the methods section. Test performance was calculated for all categories assuming that all high-risk women will receive treatment, and that any intermediate-risk women with disease will be diagnosed during follow-up and receive treatment, without further exploring the optimal management of this group.

### Dual result approach

In these seven strategies (F-L) only high and low risk groups were identified with no intermediate risk group. The sequence of tests did not influence the performance of the strategy, but the positivity rate of the first test reflects the number of participants who needed a second test, which will influence costs. (Table 2). In strategy L all hrHPV positive samples underwent partial genotyping (PGT) for 16/18 as is built into this HPV test; and those not positive for these two highest risk types, were reflexed to cytology; thus the “second test” was essentially a combination of PGT and cytology.

**Table 2:** Performance of combination test strategies using a dual result approach (high- and low-risk result) among HIV-positive and HIV-negative cohorts

HPW: HIV-positive women (n = 456)	Test positivity rate			Histology diagnosis used to calculate test performance			
Test 1 (threshold); Test 2 (threshold)	Primary test positive: Test 1 as primary	Primary test positive: Test 2 as primary	Dual test positive: High-risk group	CIN3 + prevalence: 106/456 (23.3%) ICC prevalence: 13/456 (2.9%)		CIN2 + prevalence: 203/456 (44.5%)	
Performance indicators	Reflex to second test %	Reflex to second test %	Refer to treatment %	Sensitivity %	NPV %	Specificity %	PPV %
F: Visual inspection (VIA?); Cytology (ASCUS +)	47.8	39.9	28.3	64.2	88.4	90.9	82.2
G: Visual inspection (VIA?); Cytology (LSIL +)	47.8	33.4	25.0	62.3	88.3	93.7	86.0
H: Visual inspection (VIA?); hrHPV (any)	47.8	48.5	29.8	67.0	89.1	91.7	84.6
I: hrHPV (any); Cytology (ASCUS +)	48.5	39.9	31.4	70.8	90.1	90.9	83.9
J: hrHPV (any); Cytology (LSIL +)	48.5	33.4	28.1	68.9	90.0	92.5	85.2
K: hrHPV (any); HPV (16/18)	48.5	N/A	17.1	37.7	82.5	96.4	88.5
L: hrHPV (any); HPV16/18 OR Cytology (ASCUS +) *	48.5	N/A	34.7	73.6	90.6	89.7	83.5

HIV-negative women, HPW (n = 648)	Test positivity rate			Histology diagnosis used to calculate test performance			
Test 1 (threshold); Test 2 (threshold)	Primary test positive: Test 1 as primary	Primary test positive: Test 2 as primary	Dual test positive: High-risk group	CIN3 + prevalence: 67/648 (10.3%); ICC prevalence: 9/648 (1.4%)		CIN2 + prevalence: 164/648 (25.3%)	
Performance indicators	Reflex to second test %	Reflex to second test %	Refer to treatment %	Sensitivity %	NPV %	Specificity %	PPV %
F: Visual inspection (VIA?); Cytology (ASCUS +)	18.7	17.0	8.2	42.4	93.6	96.9	71.7
G: Visual inspection (VIA?); Cytology (LSIL +)	18.7	8.0	5.3	30.3	92.5	98.6	79.4
H: Visual inspection (VIA?); hrHPV (any)	18.7	23.5	7.4	43.9	93.8	97.7	77.1
I: hrHPV (any); Cytology (ASCUS +)	23.5	17.0	10.2	50.0	94.3	96.5	74.2
J: hrHPV (any); Cytology (LSIL +)	23.5	8.0	5.7	33.3	92.8	98.4	78.4
K: hrHPV (any); HPV (16/18)	23.5	N/A	7.6	34.9	92.8	96.7	67.4
L: hrHPV (any); HPV16/18 OR Cytology (ASCUS +)*	23.5	N/A	13.9	59.1	95.2	93.6	65.6

CIN2 +  = cervical squamous intraepithelial neoplasia grade 2 or worse;

CIN3 +  = cervical squamous intraepithelial neoplasia grade 3 or worse;

ICC = invasive cervical cancer;

VIA? = visual inspection with acetic acid of uncertain or worse;

VILI? = visual inspection with Lugol’s iodine of uncertain or worse;

ASCUS = atypical squamous cells of unknown significance or worse;

LSIL = low grade squamous intra-epithelial lesion or worse;

any = any one or more of the 14 specified high-risk HPV DNA types

16/18 = positive for HPV DNA of either HPV16 or HPV18 or both

^*^Test 2 is defined as the combination of partial genotyping (HPV16/18) and cytology (threshold ASCUS) for hrHPV positives who are not HPV16/18 positive

95% Confidence intervals are shown in the supplementary files

Strategy G, which combines VIA and cytology at LSIL threshold, had the highest specificity in both groups, but poor sensitivity among HNW. Strategy L had the highest sensitivity and best overall performance in both cohorts. Relative sensitivities of strategy L compared to strategy G were 118% (HPW) and 195% (HNW), while relative specificities were 95.7% (HPW) and 94.9% (HNW) (*p* < 0.05 for all comparisons). The strategies can therefore be considered comparable in HPW, but strategy L was superior in HNW.

In the HIV-positive cohort, combinations of hrHPV testing with cytology (I, J, L) all reached both the selected sensitivity and specificity standards, with treatment rates ranging between 28.1 and 34.7%. All these strategies performed very similar to the best single test, namely cytology (LSIL), strategy D.

In the HIV-negative cohort, the best sensitivity (59.1%) was reached in strategy L, while all others had a sensitivity of 50% or below*.* Relative sensitivity of strategy L (best dual strategy) compared to hrHPV (any) (best single test)(E), was 86.7%, and relative specificity was 110%. Importantly however, treatment referrals were much lower (13.9% vs. 23.5%) in strategy L.

### Triple result approach

The performance of 12 strategies resulting in high-risk (double positive), intermediate-risk (only primary test positive), and low-risk groups (primary test negative), were calculated as described above; combinations M to Q each has two strategic options based on sequence of testing, R and S has only one option. (Table 3).

**Table 3:** Performance of combination test strategies using a triple result approach (high-, intermediate-, and low-risk results) among HIV-positive and HIV-negative cohorts

HPW: HIV-positive women (n = 456)	Screening sequence 1: Test 1 as primary and Test 2 as secondary test			Screening sequence 2: Test 2 as primary and Test 1 as secondary test				Performance of strategy	
Test 1 (threshold); Test 2 (threshold)	Single test positive: Intermediate-risk group	CIN3 + prevalence: 106/456 (23.3%); ICC prevalence: 13/456 (2.9%)		Single test positive: Intermediate-risk group	CIN3 + prevalence: 106/456 (23.3%); ICC prevalence: 13/456 (2.9%)		Dual test positive: High-risk group	CIN2 + prevalence: 203/456 (44.5%)	
Performance indicators	Recall for follow-up %	Sensitivity %	NPV %	Recall for follow-up %	Sensitivity %	NPV %	Refer to treatment %	Specificity %	PPV %
M: Visual inspection (VIA?); Cytology (ASCUS +)	19.5	75.5	89.1	11.6	75.5	90.5	28.3	90.9	82.2
N: Visual inspection (VIA?); Cytology (LSIL +)	22.8	75.5	89.1	7.5	71.2	90.3	25.0	93.7	86.0
O: Visual inspection (VIA?); hrHPV (any)	18.0	75.5	89.1	18.6	82.1	91.9	29.8	91.7	84.6
P: hrHPV (any); Cytology (ASCUS +)	17.1	82.1	91.9	8.6	75.5	90.5	31.4	90.9	83.9
Q: hrHPV (any); Cytology (LSIL +)	20.4	82.1	91.9	4.4	71.2	90.3	28.1	92.5	85.2
R: hrHPV (any); HPV(16/18)	31.4	82.1	91.9	N/A	N/A	N/A	17.1	96.4	88.5
S: hrHPV (any); HPV(16/18) OR Cytology (ASCUS +)	13.8	82.1	91.9	N/A	N/A	N/A	34.7	89.7	83.5

HNW: HIV-negative women (n = 648)	Screening sequence 1: Test 1 as primary and Test 2 as secondary test			Screening sequence 2: Test 2 as primary and Test 1 as secondary test				Performance of strategy	
Test 1 (threshold); Test 2 (threshold)	Single test positive: Intermediate-risk group	CIN3 + prevalence: 67/648 (10.3%); ICC prevalence: 9/648 (1.4%)		Single test positive: Intermediate-risk group	CIN3 + prevalence: 67/648 (10.3%); ICC prevalence: 9/648 (1.4%)		Dual test positive: High-risk group	CIN2 + prevalence: 164/648 (25.3%)	
Performance indicators	Recall for follow-up %	Sensitivity %	NPV %	Recall for follow-up %	Sensitivity %	NPV %	Refer to treatment %	Specificity %	PPV %
M: Visual inspection (VIA?); Cytology (ASCUS +)	10.7	50.0	93.7	8.8	59.1	95.0	8.2	96.9	71.7
N: Visual inspection (VIA?); Cytology (LSIL +)	13.5	50.0	93.7	2.8	35.9	93.1	5.3	98.6	79.4
O: Visual inspection (VIA?); hrHPV(any)	11.3	50.0	93.7	16.1	68.2	95.8	7.4	97.7	77.1
P: hrHPV (any); Cytology (ASCUS +)	13.3	68.2	95.8	6.8	59.1	95.0	10.2	96.5	74.2
Q: hrHPV (any); Cytology(LSIL +)	17.8	68.2	95.8	2.2	35.9	93.1	5.7	98.4	78.4
R: hrHPV (any); HPV(16/18)	15.9	68.2	95.8	N/A	N/A	N/A	7.6	96.7	67.4
S: hrHPV (any); HPV (16/18) OR Cytology (ASCUS +)	9.6	68.2	95.8	N/A	N/A	N/A	13.9	93.6	65.6

Intermediate risk group = single test positive, recall for follow-up;

High risk group = double test positive, for treatment;

Low risk group = double test negative, for routine screening interval;

CIN2 +  = cervical squamous intraepithelial neoplasia grade 2 or worse;

CIN3 +  = cervical squamous intraepithelial neoplasia grade 3 or worse;

ICC = invasive cervical cancer;

VIA? = visual inspection with acetic acid of uncertain or worse;

VILI? = visual inspection with Lugol’s iodine of uncertain or worse;

ASCUS = atypical squamous cells of unknown significance or worse;

LSIL = low grade squamous intra-epithelial lesion or worse;

any = any one or more of the 14 specified high-risk HPV DNA types;

16/18 = positive for HPV DNA of either HPV16 or HPV18 or both

95% Confidence intervals are shown in the supplementary files

In general, screening with visual inspection (M1, N1, O1) resulted in larger intermediate risk groups than cytology screening (M2,N2,P2); all these options had acceptable sensitivity among HPW, but poor among HNW. In both HIV-groups, primary screening with hrHPV (any) had the best sensitivity and all secondary test options (P1, Q1, R, S) resulted in excellent specificity; the selected secondary test determined the relative sizes of treatment versus follow-up groups. Using the combination of HPV16/18 and cytology (ASCUS +) as secondary test (strategy S), had the largest treatment and the smallest follow-up groups.

In the HIV-positive cohort, the sensitivity and specificity of all strategies (M-S) reached acceptability. Using hrHPV (any) as primary and hrHPV (16/18) as secondary tests (strategy R) resulted in the best sensitivity, best specificity, smallest treatment group (17.1%), but the largest follow-up group. Of those strategies combining visual inspection and cytology, strategy N1 had the smallest treatment group and an acceptable follow-up group; 47.8% would need cytology.

In the HIV-negative cohort, all strategies had excellent specificity, but only strategies starting with hrHPV (any) (O2, P1, Q1, R, S) reached the sensitivity standard. All of VIA, cytology(LSIL) and HPV(16/18) (O2,Q1,R) had comparable accuracy when used as secondary tests, with treatment groups between 5.7 and 7.6% and follow-up groups between 15.9 and 17.8%.

## Discussion

### Epidemiology

This real-world study group was selected to be representative of the local public sector cervical cancer screening population in terms of age range, screening history, and the HIV-treatment and disease control of HPW. The high cervical disease and hrHPV prevalence is typical of the South African public health service population; underlying causes and implications were discussed before [26]. Similar high disease prevalence is reported in several other subpopulations in the region and in sub-Saharan Africa [14, 30–34]. Contrary to this high disease burden, comparative study populations from the Global North are typically heavily pre-screened and many study groups are enriched with referral populations to address a low disease prevalence [10–12, 20, 35, 36].

In addition to true epidemiological differences, the majority of screening studies omit histology in screen-negatives and report disease prevalence based on the assumption that negative screening tests are true negatives [10–12, 23]. In this project, about half of screen-negative women has biopsy data, with enough unexpected positive histology results to influence the calculated test performance. Further investigation of the unexpected high number of CIN2 + histology is underway in the form of histology review, immunohistochemistry, and extended genotyping to understand the role of non-high-risk types, and correlation with methylation markers.

### Screening strategy criteria

As far as we could establish universal criteria to evaluate, compare and interpret test performance and select screening strategies do not exist – usually the “best” option is selected per population [37]. The ideal balance of sensitivity and specificity depends on the management algorithm, available health infrastructure, screening frequency, tolerance for missed cases and overtreatment, etc. In South Africa, and similar societies desperate to address high disease burdens, the best possible sensitivity is needed due to long screening intervals, but in test-and-treat programmes high test specificity is also needed to limit overtreatment.

In addition to having excellent test performance, the strategy should be as simple as possible; the simplest is a dual-result approach where positives are treated without further testing and negatives are referred back to the next screening round. If that is not possible, the size of the intermediate-risk group should be as small as possible. Similarly, having a universally applicable screening strategy is preferable, or at least a universal screening test, with the option of different management algorithms [22]. We chose as a standard of acceptability the best universally achievable sensitivity (68% for CIN3 +), and to limit overtreatment to 15% (specificity of 85% for < CIN2 +).

### Single test strategies

The sensitivity of cytology and visual inspection among HPW was significantly better than among HNW, as has also been reported by many others [14, 15, 17, 31]. In HPW these tests performed similar or even better than found in other studies from China, Sub-Saharan Africa, and South Africa, possibly partially explained by the fact that most HPW in this study were screened at a single facility and by a single, experienced, and well-trained nurse colposcopist [14, 15, 17]. In spite of our well-established cytology service, the sensitivity of cytology among HNW was poor, and only cytology at the low cut-off of “ASCUS + ” came near acceptability with a sensitivity of 59%. Visual inspection among HNW was done at several study centres by different investigators and performed poorly.

As expected, and found by other researchers, hrHPV(any) had the highest overall sensitivity, which was better among HPW than HNW, while the specificity was higher among HNW [17, 18, 38]. Immediate referral of women with HPV types 16/18 (and 45) is already widely recommended [12, 30, 39]. Here we showed that the hrHPV (any) had sufficient specificity for immediate treatment without further genotyping in HNW but not HPW. In this study we could, however, not duplicate the widely reported ultra-high sensitivity of HPV-tests in either of the cohorts [14, 40, 41]. Probable contributing factors include differences in epidemiology, dysplasia associated with low-risk HPV types, underdiagnosis in other studies due to lack of histology among screen-negatives and histological overdiagnosis in the current study. Further investigation of hrHPV-negative CIN2 + lesions are underway.

Unfortunately, among single test strategies with sensitivity near the selected standard of 68%, the best universally achievable specificity was 75% for < CIN2 (25% overtreatment). South Africa and other nations with large HIV-positive populations, are therefore forced to consider combination test strategies in order to achieve a specificity of 85% or to have separate screening tests for HPW vs. HNW.

### WHO recommendations

When considering the recommendations made in the WHO 2021 guidelines [13], this study confirms recommendations nr. 1, 2 & 3 for general populations or HNW, namely that they should be screened with HPV-testing rather than VIA or cytology (poor sensitivity) and that a second test is not strictly needed before treatment (good specificity). The data presented here, however, showed high referral numbers based on hrHPV only, and that a second test would reduce referral and treatment burdens. On the other hand, recommendation nr.21, stating that HPW should be screened with an HPV-test in favour of cytology, is not supported by the current study. In the HIV-positive cohort cytology performed better than HPV-screening, as was also found by others [15, 17, 18]. Our data and calculations support the recommendation (nr. 22) that HPV-screening requires a second test when used in HPW (due to poor specificity).

### Dual result combination strategies

In HPW all dual result strategies which combine cytology and hrHPV testing, showed acceptable and comparable test performance, with treatment groups from 28.1% to 34.7%. These performance and referral rates were similar to the best single test strategy cytology (LSIL), but better than hrHPV(all) alone.

In HNW the best dual result combination strategy was HPV-screening, followed by partial genotyping and cytology for non-16/18 HPV-positives (L) resulting in a treatment group of 13.9%. It was superior to the best single test strategy (hrHPV (all)) due to a smaller treatment group and better specificity. This strategy also had an excellent performance in HPW, which is in accordance with other reports [17, 21]. We found that this was the best dual result strategy to implement universally.

### Triple results combination strategies

Among HPW all calculated triple result strategies reached both acceptability standards with similar test performance, but differences in treatment and follow-up group sizes. Our findings support the WHO recommendation (nr. 23) that partial genotyping, visual inspection, and cytology are all valid and similarly performing triage tests after hrHPV-screening (our strategies O2, R, P), but also validates cytology as primary test in a combination approach. Among HNW only combination test strategies starting with hrHPV (all) were acceptable and visual inspection, cytology, and partial genotyping with/without cytology performed well as secondary tests.

For universal implementation, the best triple result strategy appears to be the same combination as described as the preferred dual result combination strategy (L), but now with an intermediate-risk category (S). It results in the smallest intermediate risk group which consist of non-16/18 hrHPV positive women without cytologic abnormalities. The identification and management of the intermediate-risk group will increase the sensitivity by about 10 percentage points (from 73.6 to 82.1% in HPW; from 59.1 to 68.2% in HNW). Similar to the current study, others have also shown that treatment of HPV 16/18 combined with triage of other HPV types achieves an excellent balance of sensitivity and specificity among HPW [42]. Among HNW, follow-up of the intermediate risk group is recommended due to low sensitivity [43]. This strategy has high treatment numbers and is expected to have the fastest and biggest impact on invasive cancer prevalence, as treatment of all HPV16/18 should prevent at least 2/3 of cancer cases, and the addition of cytology for the other hrHPV positives will improve on this even without effective recall [44].

When calculating test performance, the allowance for an intermediate-risk group preserves the sensitivity of the initial test and increases specificity by refining the treatment group. Planning and implementing effective management for these intermediate-risk women will undoubtedly be challenging and expensive but is essential to realise the test performance quoted here. In the absence of effective recall, it is advisable to select a dual result strategy with a high specificity and smaller treatment groups, without the false promise of high sensitivity.

### Single laboratory test

Using only one laboratory test, the best universal approach in this study was screening with hrHPV, followed by built-in partial genotyping as secondary test. HPV16/18 positives are referred to treatment, while non-16/18 positives are managed as an intermediate-risk group (R). We did not further investigate different management options for this latter group but presumed a zero lost-to-follow-up rate in calculating the sensitivity (increase from 37.7 to 82.1% in HPW and from 34.9% to 68.2% in HNW in comparison with strategy K). This strategy can be selected if it is expected that treatment facilities will be overwhelmed, or if capacity does not allow for the addition of cytology triage; treatment numbers will be half of that resulting from the triple result strategy which includes cytology (S) as described above.

### Place for visual inspection

The only universally acceptable strategy employing visual inspection, was to use HPV as primary test and visual inspection as secondary (O2). Employing a triple result approach, this strategy resulted in treating 29.8 and 7.4% and following 18.6 and 16.1% of HPW and HNW respectively and can be a useful cheaper strategy using a cheaper non-discriminatory HPV test without the need for cytology. Alternatively, using a dual-result approach with the same two tests, acceptable test performance can be reached without the need for intermediate-risk groups. Using primary HPV-screening universally as primary test, it can be followed by two different strategies for the two HIV-subgroups: All hrHPW-positive HNW are directly referred for treatment (E), but positive HPW are called for VI, only double-positives are referred for treatment (H2). VILI should be preferred above VIA here due to superior sensitivity.

### Relevance for screening policy

We previously discussed the high disease prevalence in our country which was confirmed here [26, 45]. While our study investigated different screening algorithms, it is acknowledged that the efficacy of secondary prevention will depend on improving on the current low treatment rates [46].

## Conclusion

This cross-sectional cohort screening study showed unusually high prevalence of screen positivity and histology proven disease for both study groups, confirming the need to re-calculate the performance of screening tests to enable choice of strategy, and calculation of budget and infrastructure needs.

The performance of various screening strategies was calculated and tested considering acceptability standards, universal applicability, and the size of the intermediate-risk vs. treatment groups. Results were markedly different between HIV-positive and HIV-negative women and incomparable with published reports from other study populations. Several findings from this study will be significant for future screening programmes.

Firstly, universal cytology screening cannot be supported, as it is a low-performance test among the HNW or general populations. Secondly, there is no remaining role for visual inspection as primary test as the specificity was too low for treatment purposes. Thirdly, we conclude that primary hrHPV screening is the only screening method with appropriate sensitivity to be used universally and that the built-in HPV16/18 test is sufficient as secondary test to indicate need for treatment in both HIV-subgroups. Using different algorithms to manage those with non16/18 HPV, HNW need increased surveillance (strategy R), but HPW do not (strategy L). The addition of cytology (strategy S) is an option for both groups which significantly reduces the follow-up burden by increasing the treatment group.

### Strengths and limitations

This is one of few studies to report screening test performance on both HIV-positive and negative women from the same population. The large percentage of women who had biopsies and the calculation of verification bias adjusted (VBA) histology data for all disease categories in the remaining women are important strengths. The calculated test performance and positivity rates allows for calculation of budget and service needs for a variety of single and combination test strategies.

While the study contributes to fill a data gap for countries similar to South Africa, the findings will only be valid and applicable for populations with similar demographical data. The relatively small sample size was sufficient for our calculations in view of the high disease prevalence, but it limits accurate sub-analyses. This study did not address long term prediction or screening interval, but a longitudinal follow-up study is planned to address this question.

### Supplementary Information

Supplementary file1 (DOCX 37 KB)

## Data Availability

Cited references and supplementary data files provide data in support of the findings and further data will be made available upon reasonable request.

## Abbreviations

ARV: Antiretroviral
ASCUS: Atypical squamous cells of undetermined significance
AUC: Area under the curve
CI: Confidence interval
CIN: Cervical intraepithelial neoplasia
DiaVACCS: Diagnosis in vaccine and cervical cancer screen
DNA: Deoxyribonucleic acid
HIV: Human immunodeficiency virus
HNW: HIV-negative women
HPV: Human papillomavirus
hrHPV/HPV(hr): High-risk human papillomavirus
HPW: HIV-positive women
ICC: Invasive cervical cancer
LLETZ: Large loop excision of the transformation zone
LSIL: Low-grade squamous intraepithelial lesion
NPV: Negative predictive value
PPV: Positive predictive value
ROC: Receiver operator characteristic
VACCS: Vaccine and cervical cancer screen
VBA: Verification biased adjusted
VIA: Visual inspection using 3–5% acetic acid
VILI: Visual inspection using Lugol’s iodine solution
WHO: World Health Organization
