# Supplementary material for: The performance of single and combination test strategies using visual inspection, cytology, high-risk HPV DNA and HPV16/18 to screen South African women with and without HIV-infection
Source: Infect Agent Cancer. 2024 May 9;19:22. doi: 10.1186/s13027-024-00586-3 (PMC11084067; doi:10.1186/s13027-024-00586-3)
Supplement: Supplementary file 1 — Supplementary file1 (DOCX 37 KB) [file 13027_2024_586_MOESM1_ESM.docx]

**Table 1. Performance of single test strategies to predict CIN2+ and CIN3+ histology among HIV positive and HIV negative cohorts (with 95% CI)**

| **HIV-positive women, HPW (n=456)** | | **Histology diagnosis used to calculate test performance** | | | | | | | | |
| --- | --- | --- | --- | --- | --- | --- | --- | --- | --- | --- |
| **Test (threshold)** | **Positivity rate %**  [95% CI] | **CIN2+ histology**  **CIN2+ Prevalence: 203/456 (44.5%)** | | | | **CIN3+ histology**  **CIN3+ Prevalence: 106/456 (23.3%);**  **ICC prevalence: 13/456 (2.9%)** | | | | |
| **Performance indicators** | **Referral to treatment** | **Sensitivity %**  [95% CI] | **Specificity %**  [95% CI] | **PPV %**  [95% CI] | **NPV %**  [95% CI] | **Sensitivity %**  [95% CI] | **Specificity %**  [95% CI] | **PPV %**  [95% CI] | **NPV %**  [95% CI] |  |
| **A: Visual inspection (VIA?)** | 47.8  [43.2-52.4] | 66.5  [60.0-73.0] | 67.2  [61.4-73.0] | 61.9  [55.4-68.4] | 71.4  [65.7-77.2] | 75.5  [67.2-86.8] | 60.6  [55.4-65.7] | 36.7  [30.3-43.1] | 89.1  [85.1-93.0] |  |
| **B: Visual inspection (VILI?)** | 50.4  [45.8-55.0] | 67.0  [60.5-73.5] | 62.9  [56.9-68.8] | 59.1  [52.7-65.5] | 70.4  [64.4-76.3] | 76.4  [68.3-84.6] | 57.4  [52.2-62.6] | 35.2  [29.0-41.4] | 88.9  [84.8-93.1] |  |
| **C: Cytology(ASCUS)** | 39.9  [35.4-44.1] | 63.6  [56.9-70.2] | 79.1  [74.0-84.1] | 70.9  [64.2-77.5] | 73.0  [66.5-79.5] | 75.5  [67.2-83.8] | 70.9  [66.1-75.6] | 44.0  [36.7-51.2] | 90.5  [87.0-94.0] |  |
| **D: Cytology(LSIL)** | 33.4  [29.0-37.8] | 55.7  [48.8-62.6] | 90.5  [86.8-94.2] | 83.0  [76.6-89.4] | 71.1  [63.4-78.8] | 71.2  [62.3-80.0] | 82.0  [77.9-86.1] | 54.8  [46.3-63.3] | 90.3  [86.9-93.6] |  |
| **E: hrHPV(any)** | 48.5  [43.9-53.1] | 78.8  [73.2-84.5] | 75.9  [70.6-81.2] | 72.4  [66.5-78.3] | 81.7  [76.6-86.8] | 82.1  [74.7-89.5] | 61.7  [56.6-66.8] | 39.4  [32.9-45.8] | 91.9  [88.4-95.4] |  |
| **HIV-negative women, HNW (n=648)** | | **Histology diagnosis used to calculate test performance** | | | | | | | | |
| **Test (threshold)** | **Positivity rate %**  [95% CI] | **CIN2+ prevalence: 164/648 (25.3%)** | | | | **CIN3+ prevalence: 67/648 (10.3%);**  **ICC prevalence: 9/648 (1.4%)** | | | | |
| **Performance indicators** | **Referral to treatment** | **Sensitivity %**  [95% CI] | **Specificity %**  [95% CI] | **PPV %**  [95% CI] | **NPV %**  [95% CI] | **Sensitivity %**  [95% CI] | **Specificity %**  [95% CI] | **PPV %**  [95% CI] | **NPV %**  [95% CI] |  |
| **A: Visual inspection (VIA?)** | 18.7  **[15.7-21.7]** | 35.6  [28.2-43.0] | 87.0  [84.0-90.0] | 47.9  [38.9-56.9] | 80.0  [76.6-83.5] | 50.0  [37.7-62.3] | 84.9  [81.9-87.8] | 27.3  [19.3-35.3] | 93.7  [91.7-95.8] |  |
| **B: Visual inspection (VILI?)** | **20.9**  **[17.7-24.0]** | 35.6  [28.2-43.0] | 84.1  [80.8-87.4] | 43.0  [34.5-51.4] | 79.5  [72.6-86.4] | 51.5  [39.2-63.8] | 82.6  [79.5-85.7] | 25.2  [17.8-32.6] | 93.8  [91.7-95.9] |  |
| **C: Cytology(ASCUS)** | **17.0**  **[14.1-20.0]** | 41.7  [34.1-49.3] | 91.3  [88.8-93.9] | 61.8  [52.6-71.0] | 82.3  [75.1-89.6] | 59.1  [47.0-71.2] | 87.8  [85.1-90.5] | 35.5  [26.4-44.5] | 95.0  [93.1-96.8] |  |
| **D: Cytology(LSIL)** | **8.0**  **[5.9-10.1]** | 20.1  [13.9-26.4] | 97.5  [96.1-98.9] | 72.7  [59.2-86.3] | 78.7  [66.3-91.2] | 35.9  [24.0-47.9] | 96.4  [94.8-97.9] | 52.3  [37.1-67.5] | 93.1  [91.1-95.2] |  |
| **E: hrHPV(any)** | **23.5**  **[20.2-26.7]** | 49.1  [41.4-56.8] | 85.2  [82.0-88.3] | 52.6  [44.6-60.6] | 83.3  [77.3-89.3] | 68.2  [56.7-79.3] | 81.6  [78.5-84.8] | 29.6  [22.3-36.9] | 95.8  [94.0-97.5] |  |

CIN2+ = cervical squamous intraepithelial neoplasia grade 2 or worse; CIN3+ = cervical squamous intraepithelial neoplasia grade 3 or worse; ICC = invasive cervical cancer; VIA? = visual inspection with acetic acid uncertain or worse; VILI? = visual inspection with Lugol’s iodine uncertain or worse; ASCUS+ = atypical squamous cells of unknown significance or worse result; LSIL+ = low grade squamous intra-epithelial lesion or worse result; any = any of the 14 specified high risk HPV DNA types; 95% CI = 95% Confidence interval

**Table 2: Performance of combination test strategies using a dual result approach (high- and low-risk result) among HIV-positive and HIV-negative cohorts (with 95% CI).**

| **HIV-positive women, HPW (n=456)** | **Test positivity rate** | | | **Histology diagnosis used to calculate test performance** | | | |
| --- | --- | --- | --- | --- | --- | --- | --- |
| **Test 1 (threshold);**  **Test 2 (threshold)** | **Primary test positive:**  **Test 1 as primary** | **Primary test positive:**  **Test 2 as primary** | **Dual test positive:**  **High-risk group** | **CIN3+ prevalence: 106/456 (23.3%)**  **ICC prevalence: 13/456 (2.9%)** | | **CIN2+ prevalence: 203/456 (44.5%)** | |
| **Performance indicators** | **Reflex to second test %**  [95% CI] | **Reflex to second test %**  [95% CI] | **Refer to treatment %**  [95% CI] | **Sensitivity %**  [95% CI] | **NPV %**  [95% CI] | **Specificity %**  [95% CI] | **PPV %**  [95% CI] |
| **F: Visual inspection (VIA?);**  **Cytology (ASCUS+)** | 47.8  [43.2-52.4] | 39.9  [35.4-44.1] | 28.3  [24.1-32.4] | 64.2  [54.9-73.4] | 88.4  [84.9-91.9] | 90.9  [87.4-94.5] | 82.2  [75.5-88.8] |
| **G: Visual inspection (VIA?);**  **Cytology (LSIL+)** | 47.8  [43.2-52.4] | 33.4  [29.0-37.8] | 25.0  [21.0-29.0] | 62.3  [52.9-71.6] | 88.3  [84.9-91.7] | 93.7  [90.7-96.7] | 86.0  [79.5-92.4] |
| **H: Visual inspection (VIA?);**  **hrHPV (any)** | 47.8  [43.2-52.4] | 48.5  [43.9-53.1] | 29.8  [25.6-34.0] | 67.0  [57.9-76.0] | 89.1  [85.6-92.5] | 91.7  [88.3-95.1] | 84.6  [78.4-9.7] |
| **I: hrHPV (any);**  **Cytology (ASCUS+)** | 48.5  [43.9-53.1] | 39.9  [35.4-44.1] | 31.4  [27.1-35.6] | 70.8  [62.0-79.5] | 90.1  [86.8-93.4] | 90.9  [87.4-94.5] | 83.9  [77.8-90.0] |
| **J: hrHPV (any);**  **Cytology (LSIL+)** | 48.5  [43.9-53.1] | 33.4  [29.0-37.8] | 28.1  [23.9-32.2] | 68.9  [60.0-77.8] | 90.0  [86.7-93.2] | 92.5  [89.2-95.8] | 85.2  [78.9-91.4] |
| **K: hrHPV (any);**  **HPV (16/18)** | 48.5  [43.9-53.1] | N/A | 17.1  [13.6-20.6] | 37.7  [28.4-47.1] | 82.5  [78.7-86.4] | 96.4  [94.2-98.4] | 88.5  [81.3-95.7] |
| **L: hrHPV (any); HPV16/18 OR Cytology (ASCUS+) *** | 48.5  [43.9-53.1] | N/A | 34.7  [30.3-39.0] | 73.6  [65.1-82.1] | 90.6  [87.3-93.9] | 89.7  [86.0-93.5] | 83.5  [77.7-89.4] |
| **HIV-negative women, HPW (n=648)** | **Test positivity rate** | | | **Histology diagnosis used to calculate test performance** | | | |
| **Test 1 (threshold);**  **Test 2 (threshold)** | **Primary test positive:**  **Test 1 as primary** | **Primary test positive:**  **Test 2 as primary** | **Dual test positive:**  **High-risk group** | **CIN3+ prevalence: 67/648 (10.3%);**  **ICC prevalence: 9/648 (1.4%)** | | **CIN2+ prevalence: 164/648 (25.3%)** | |
| **Performance indicators** | **Reflex to second test %**  [95% CI] | **Reflex to second test %**  [95% CI] | **Refer to treatment %**  [95% CI] | **Sensitivity %**  [95% CI] | **NPV %**  [95% CI] | **Specificity %**  [95% CI] | **PPV %**  [95% CI] |
| **F: Visual inspection (VIA?);**  **Cytology (ASCUS+)** | 18.7  **[15.7-21.7]** | **17.0**  **[14.1-20.0]** | 8.2  [6.1-10.3] | 42.4  [30.3-54.8] | 93.6  [91.6-95.6] | 96.9  [95.4-98.5] | 71.7  [59.3-84.1] |
| **G: Visual inspection (VIA?);**  **Cytology (LSIL+)** | 18.7  **[15.7-21.7]** | **8.0**  **[5.9-10.1]** | 5.3  [3.5-7.0] | 30.3  [19.0-41.6] | 92.5  [90.4-54.6] | 98.6  [97.5-99.6] | 79.4  [65.3-93.5] |
| **H: Visual inspection (VIA?);**  **hrHPV (any)** | 18.7  **[15.7-21.7]** | **23.5**  **[20.2-26.7]** | 7.4  [5.4-9.4] | 43.9  [31.7-56.1] | 93.8  [91.9-95.8] | 97.7  [96.4-99.1] | 77.1  [64.9-89.3] |
| **I: hrHPV (any);**  **Cytology (ASCUS+)** | **23.5**  **[20.2-26.7]** | **17.0**  **[14.1-20.0]** | 10.2  [7.9-12.5] | 50.0  [37.7-62.3] | 94.3  [92.5-96.2] | 96.5  [94.9-98.1] | 74.2  [63.5-85.0] |
| **J: hrHPV (any);**  **Cytology (LSIL+)** | **23.5**  **[20.2-26.7]** | **8.0**  **[5.9-10.1]** | 5.7  [3.9-7.5] | 33.3  [21.7-44.9] | 92.8  [90.7-94.9] | 98.4  [97.2-99.5] | 78.4  [64.7-92.1] |
| **K: hrHPV (any);**  **HPV (16/18)** | **23.5**  **[20.2-26.7]** | N/A | 7.6  [5.5-9.6] | 34.9  [23.1-46.6] | 92.8  [90.8-94.9] | 96.7  [95.1-98.3] | 67.4  [53.9-80.8] |
| **L: hrHPV (any); HPV16/18 OR Cytology (ASCUS+) *** | **23.5**  **[20.2-26.7]** | N/A | 13.9  [11.2-16.6] | 59.1  [47.0-71.2] | 95.2  [93.4.-97.0] | 93.6  [91.4-95.8] | 65.6  [55.6-75.5] |

CIN2+ = cervical squamous intraepithelial neoplasia grade 2 or worse; CIN3+ = cervical squamous intraepithelial neoplasia grade 3 or worse; ICC = invasive cervical cancer; VIA? = visual inspection with acetic acid with result of uncertain or worse; ASCUS+ = atypical squamous cells of unknown significance or worse result; LSIL+ = low grade squamous intra-epithelial lesion or worse result; any = any of the 14 specified high risk HPV DNA types; 16/18 = positive for HPV DNA of either HPV16 or HPV18 or both

95% CI = 95% Confidence interval

*Test 2 is defined as the combination of partial genotyping (HPV16/18) and cytology (threshold ASCUS) for hrHPV positives who are not HPV16/18 positive.

**Table 3: Performance of combination test strategies using a triple result approach (high-, intermediate- and low-risk results) among HIV-positive and HIV-negative cohorts (with 95% CI)**

| **HIV-positive women, HPW (n=456)** | **Screening sequence 1:**  **Test 1 as primary and Test 2 as secondary test** | | | **Screening sequence 2:**  **Test 2 as primary and Test 1 as secondary test** | | | **Performance of strategy** | | |
| --- | --- | --- | --- | --- | --- | --- | --- | --- | --- |
| **Test 1 (threshold);**  **Test 2 (threshold)** | **Single test positive: Intermediate-risk group** | **CIN3+ prevalence: 106/456 (23.3%);**  **ICC prevalence: 13/456 (2.9%)** | | **Single test positive: Intermediate-risk group** | **CIN3+ prevalence: 106/456 (23.3%);**  **ICC prevalence: 13/456 (2.9%)** | | **Dual test positive: High-risk group** | **CIN2+ prevalence:**  **203/456 (44.5%)** | |
| **Performance indicators** | **Recall for follow-up**  **%**  **[95% CI]** | **Sensitivity**  **%**  **[95% CI]** | **NPV**  **%**  **[95% CI]** | **Recall for**  **follow-up %**  **[95% CI]** | **Sensitivity**  **%**  **[95% CI]** | **NPV**  **%**  **[95% CI]** | **Refer to treatment**  **%**  **[95% CI]** | **Specificity**  **%**  **[95% CI]** | **PPV**  **%**  **[95% CI]** |
| **M: Visual inspection(VIA?); Cytology(ASCUS+)** | 19.5  [15.7 to 24.0] | 75.5  [67.2-86.8] | 89.1  [85.1-93.0] | 11.6  [8.7-15.2] | 75.5  [67.2-83.8] | 90.5  [87.0-94.0 | 28.3  [24.1-32.4] | 90.9  [87.4-94.5] | 82.2  [75.5-88.8] |
| **N: Visual inspection(VIA?); Cytology (LSIL+)** | 22.8  [18.6-27.6] | 75.5  [67.2-86.8] | 89.1  [85.1-93.0] | 7.5  [5.2-10.4] | 71.2  [62.3-80.0] | 90.3  [86.9-93.6] | 25.0  [21.0-30.0] | 93.7  [90.7-96.7] | 86.0  [79.5-92.4] |
| **O: Visual inspection(VIA?);**  **hrHPV (any)** | 18.0  [14.3-22.3] | 75.5  [67.2-86.8] | 89.1  [85.1-93.0] | 18.6  [14.9-23.1] | 82.1  [74.7-89.5] | 91.9  [88.4-95.4] | 29.8  [25.6-34.0] | 91.7  [88.3-95.1] | 84.6  [78.4-9.7] |
| **P: hrHPV(any);**  **Cytology(ASCUS+)** | 17.1  [13.5-21.4] | 82.1  [74.7-89.5] | 91.9  [88.4-95.4] | 8.6  [6.1-11.7] | 75.5  [67.2-83.8] | 90.5  [87.0-94.0 | 31.4  [27.1-35.6] | 90.9  [87.4-94.5] | 83.9  [77.8-90.0] |
| **Q: hrHPV(any);**  **Cytology(LSIL+)** | 20.4  [16.5-25.0] | 82.1  [74.7-89.5] | 91.9  [88.4-95.4] | 4.4  [2.7-6.8] | 71.2  [62.3-80.0] | 90.3  [86.9-93.6] | 28.1  [23.9-32.2] | 92.5  [89.2-95.8] | 85.2  [78.9-91.4] |
| **R: hrHPV(any);**  **HPV(16/18)** | 31.4  [26.4-36.9] | 82.1  [74.7-89.5] | 91.9  [88.4-95.4] | N/A | N/A | N/A | 17.1  [13.6-20.6] | 96.4  [94.2-98.4] | 88.5  [81.3-95.7] |
| **S: hrHPV(any); HPV(16/18) OR Cytology(ASCUS+)** | 13.8  [10.6-17.7] | 82.1  [74.7-89.5] | 91.9  [88.4-95.4] | N/A | N/A | N/A | 34.7  [30.3-39.0] | 89.7  [86.0-93.5] | 83.5  [77.7-89.4] |
| **HIV-negative women, HNW (n=648)** | **Screening sequence 1:**  **Test 1 as primary and Test 2 as secondary test** | | | **Screening sequence 2:**  **Test 2 as primary and Test 1 as secondary test** | | | **Performance of strategy** | | |
| **Test 1 (threshold);**  **Test 2 (threshold)** | **Single test positive: Intermediate-risk group** | **CIN3+ prevalence: 67/648 (10.3%);**  **ICC prevalence: 9/648 (1.4%)** | | **Single test positive: Intermediate-risk group** | **CIN3+ prevalence: 67/648 (10.3%);**  **ICC prevalence: 9/648 (1.4%)** | | **Dual test positive: High-risk group** | **CIN2+ prevalence:**  **164/648 (25.3%)** | |
| **Performance indicators** | **Recall for follow-up**  **%**  **[95% CI]** | **Sensitivity**  **%**  **[95% CI]** | **NPV**  **%**  **[95% CI]** | **Recall for follow-up**  **%**  **[95% CI]** | **Sensitivity**  **%**  **[95% CI]** | **NPV**  **%**  **[95% CI]** | **Refer to treatment**  **%**  **[95% CI]** | **Specificity**  **%**  **[95% CI]** | **PPV**  **%**  **[95% CI]** |
| **M: Visual inspection(VIA?); Cytology(ASCUS+)** | 10.7  [8.3-13.5] | 50.0  [37.7-62.3] | 93.7  [91.7-95.8] | 8.8  [6.7-11.4] | 59.1  [47.0-71.2] | 95.0  [93.1-96.8] | 8.2  [6.1-10.3] | 96.9  [95.4-98.5] | 71.7  [59.3-84.1] |
| **N: Visual inspection(VIA?); Cytology(LSIL+)** | 13.5  [10.9-16.7] | 50.0  [37.7-62.3] | 93.7  [91.7-95.8] | 2.8  [1.6-4.4] | 35.9  [24.0-47.9] | 93.1  [91.1-95.2] | 5.3  [3.5-7.0] | 98.6  [97.5-99.6] | 79.4  [65.3-93.5] |
| **O: Visual inspection(VIA?);**  **hrHPV(any)** | 11.3  [8.8-14.2] | 50.0  [37.7-62.3] | 93.7  [91.7-95.8] | 16.1  [13.1-19.5] | 68.2  [56.7-79.3] | 95.8  [94.0-97.5] | 7.4  [5.4-9.4] | 97.7  [96.4-99.1] | 77.1  [64.9-89.3] |
| **P: hrHPV(any);**  **Cytology(ASCUS+)** | 13.3  [10.6-16.4] | 68.2  [56.7-79.3] | 95.8  [94.0-97.5] | 6.8  [4.9-9.1] | 59.1  [47.0-71.2] | 95.0  [93.1-96.8] | 10.2  [7.9-12.5] | 96.5  [94.9-98.1] | 74.2  [63.5-85.0] |
| **Q: hrHPV(any);**  **Cytology(LSIL+)** | 17.8  [14.7-21.3] | 68.2  [56.7-79.3] | 95.8  [94.0-97.5] | 2.2  [1.2-3.6] | 35.9  [24.0-47.9] | 93.1  [91.1-95.2] | 5.7  [3.9-7.5] | 98.4  [97.2-99.5] | 78.4  [64.7-92.1] |
| **R: hrHPV(any);**  **HPV(16/18)** | 15.9  [13.0-19.3] | 68.2  [56.7-79.3] | 95.8  [94.0-97.5] | N/A | N/A | N/A | 7.6  [5.5-9.6] | 96.7  [95.1-98.3] | 67.4  [53.9-80.8] |
| **S: hrHPV(any); HPV(16/18) OR Cytology(ASCUS+)** | 9.6  [7.3-12.3] | 68.2  [56.7-79.3] | 95.8  [94.0-97.5] | N/A | N/A | N/A | 13.9  [11.2-16.6] | 93.6  [91.4-95.8] | 65.6  [55.6-75.5] |

Intermediate risk group = single test positive, recall for follow-up; High risk group = double test positive, for treatment; Low risk group = double test negative, for routine screening interval; CIN3+ = cervical squamous intraepithelial neoplasia grade 3 or worse; CIN2+ = cervical squamous intraepithelial neoplasia grade 2 or worse; VIA? = visual inspection with acetic acid with result of uncertain or worse; ASCUS+ = atypical squamous cells of unknown significance or worse result; LSIL+ = low grade squamous intra-epithelial lesion or worse result; any = positive for any of the 14 specified high risk HPV DNA types; 16/18 = positive for HPV DNA of either HPV16 or HPV18 or both; 95% CI = 95% Confidence interval
